# Childhood vaccination against meningitis C from 2010 to 2024: a descriptive study based on the Department of Informatics of the Brazilian National Health System (DATASUS)

**DOI:** 10.31744/einstein_journal/2026AO1927

**Published:** 2026-03-27

**Authors:** Laura Beatriz Fonseca, Carlos Eduardo de Oliveira Pereira, Taísa Roberta Lopes Machado, Alisson Bruno Luzia

**Affiliations:** 1 Universidade Federal de Minas Gerais Belo Horizonte MG Brazil Universidade Federal de Minas Gerais, Belo Horizonte, MG, Brazil.; 2 Fundação Ezequiel Dias Belo Horizonte MG Brazil Fundação Ezequiel Dias, Belo Horizonte, MG, Brazil.

**Keywords:** Vaccination coverage, Vaccination hesitancy, Meningitis, Health Information Systems, Vaccination

## Abstract

**Objective:**

To analyze the correlation between vaccination coverage against meningitis C and epidemiological indicators of morbidity and mortality associated with the disease in Brazilian pediatric populations aged 3 months to 14 years.

**Methods:**

This descriptive and exploratory epidemiological study used secondary data from the National Immunization Program Information System (SI-PNI) and Notifiable Diseases Information System (SINAN). The analysis covered the period from 2010 to 2024, and examined the relationship between vaccination coverage and the number of meningitis cases and related deaths. The data included variables such as sex, age group, vaccine dose, incidence, mortality, and geographic distribution. Descriptive statistics, including calculations of proportions and rates, were performed using Microsoft Excel® (2019).

**Results:**

Following the introduction of the meningitis C vaccine in the National Immunization Program in 2010, the incidence of the disease in Brazil decreased significantly. From 2010 to 2015, the number of confirmed cases decreased by approximately 81%, reaching the lowest recorded rate in 2021 (0.02 per 100,000 inhabitants), representing a 98% reduction. However, an increase in the incidence was observed in 2022 (0.05 per 100,000 inhabitants). Although vaccination coverage exceeded 95% in 2011, it has declined in subsequent years.

**Conclusion:**

The meningitis C vaccination program has had a positive impact on public health, contributing to a marked reduction in disease cases and related deaths since its implementation. Higher vaccination coverage was associated with lower morbidity and mortality rates.

## Highlights

High vaccine coverage correlated with sharp declines in the incidence of pediatric meningitis C.

The incidence fell by 98% by 2021 following national introduction of the MenC vaccine.

Vaccine coverage dropped after 2015, aligned with the rising cases observed in 2022.

MenC vaccination significantly reduced morbidity and mortality nationwide.

## In Brief

Vaccination against meningococcal C markedly reduced cases and deaths between 2010 and 2024. The decline in coverage (worsened by the coronavirus disease 2019 pandemic) led to an increase in cases in 2022. This study highlights the effectiveness of the vaccine and the urgent need to restore coverage, particularly in vulnerable regions.

## Graphical abstract

**Figure:**
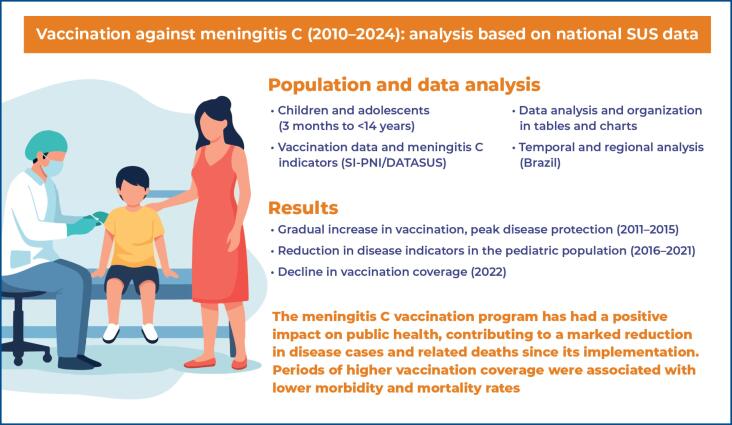

## INTRODUCTION

Bacterial meningitis, particularly that caused by the serogroup C of *Neisseria meningitidis*, represents a significant public health issue in Brazil owing to its high morbidity and mortality rates, especially among children and adolescents. Since the introduction of the conjugate vaccine against meningococcal C in Brazil's National Immunization Program (PNI) in 2010, efforts have been directed toward reducing the incidence and complications associated with this disease.^(1)^

Children and adolescents are more vulnerable to meningitis, primarily because of the immature immune system, which leads to less efficient immune responses. In addition to the acute manifestation of the disease and its high morbidity and mortality, bacterial meningitis can result in both short- and long-term complications, including a range of sequelae such as amputations, neurological deficits, development of hydrocephalus, and learning difficulties.^(2)^

The first vaccines against meningitis were developed by Emil Gotschlich in the 1970s, with the creation of the polysaccharide vaccine targeting meningococcal meningitis. However, these vaccines have notable limitations, including low immunogenicity and the inability to induce long-lasting immunological memory in children under two years of age. Consequently, polysaccharide vaccines for serogroups A, C, W, and Y have been gradually replaced with conjugate vaccines, which elicit a stronger and more effective immune response and stimulate the production of specific IgG antibodies.^(3)^ Conjugate meningococcal vaccines have demonstrated high efficacy in reducing the incidence of invasive meningitis, as confirmed in systematic reviews and meta-analyses.^(4)^

Given the predominance of serogroup C in Brazil, the meningococcal C-conjugate vaccine was incorporated into the PNI in 2010, making Brazil the first country in South America to introduce it as part of a national immunization schedule. Currently, the PNI recommends three doses of the meningococcal C conjugate vaccine for children, administered at 3, 5, and 12 months of age.^(5)^

The effectiveness of vaccination in controlling meningitis C over the years must be continually assessed using real-world data, considering the impact of epidemiological situations such as the coronavirus disease 2019 (COVID-19), vaccine hesitancy, and regional disparities in access to vaccination. Data on vaccination coverage for vaccine-preventable diseases are essential for the epidemiological analysis and evaluation of public health impacts. These data are collected by Health Information Systems (HIS), which are defined as integrated systems responsible for collecting, processing, analyzing, and transmitting the information necessary for health management.^(5,6)^

In this context, the analysis of secondary data obtained from the Department of Informatics of the Brazilian National Health System (DATASUS - *Departamento de Informação e Informática do Sistema Único de Saúde*) and the Notifiable Diseases Information System (SINAN - *Sistema de Informação de Agravos de Notificação*)^(7)^ aimed to support the optimization of vaccination strategies and the improvement of public health policies, focusing on reducing morbidity and mortality and protecting the most vulnerable populations.^(6)^ In recent years, the National Immunization Program Information System (SI-PNI),^(8)^ which monitors immunization activities in Brazil, has incorporated detailed records of administered vaccine doses, including information on the location, batch number, patient history, and tracking of vaccination coverage by region and population group.^(9,10)^

## OBJECTIVE

This study aimed to analyze the relationship between vaccination coverage against meningitis C and morbidity and mortality data related to the disease in Brazil using Health Information Systems available within the Brazilian Public Health System network, with an emphasis on the pediatric age group ranging from 3 months to 14 years.

## METHODS

### Study design

This descriptive and exploratory epidemiological study is based on secondary HIS data. It analyzes the relationship between vaccination coverage against meningitis C and disease morbidity and mortality within the context of the PNI, which incorporated this vaccine in 2010. The analysis covered the period from 2010 to 2024, considering the geographic regions and age groups within the target population.

### Population, inclusion and exclusion criteria

The study included children aged 3 months to 14 years who received the full vaccination schedule (1st dose, 2nd dose, and booster dose). Cases not laboratory-confirmed as meningitis were excluded to ensure accuracy of the analysis.

Participant selection was based on records from the SI-PNI (TabNet-DATASUS)^(8)^ complemented by the SINAN^(7)^ and Brazilian Institute of Geography and Statistics (IBGE - *Instituto Brasileiro de Geografia e Estatistica*)^(11)^ databases. The analysis focused on the correlation between official vaccination coverage and morbidity and mortality outcomes without directly addressing patients with incomplete vaccination schedules.

### Data sources and variables

Data were extracted from the SI-PNI (TabNet-DATASUS), SINAN, and VacinaBR Observatory^(12)^ with population data from the IBGE. The variables included sex, year (2010-2024), age group, number of doses administered, SINAN data on serogroup, case outcomes, and notification region.

### Data analysis

Data were organized using Microsoft Excel^®^ (2019), with descriptive charts and tables to illustrate vaccination coverage and its relationship with morbidity and mortality. The exploratory analysis included SINAN notifications considering age, demographics, and serotype prevalence by region.

The vaccination coverage data analyzed in this study refer to the official metrics calculated from the HIS of the Brazilian Public Health System (SUS - *Sistema Único de Saúde*) consolidated in the VacinaBR Observatory. The calculation of this indicator followed the standard methodology established by the PNI, in which vaccination coverage (%) is defined as the number of doses administered divided by the estimated target population multiplied by 100. The numerator corresponds to the census-based record of administered doses obtained from the SI-PNI. The denominator consists of population projections for the specific target age group stratified by the year and geographic unit, as provided by the IBGE.

## RESULTS

Data were obtained regarding vaccination coverage, the number of meningitis C cases, and deaths by age group and region. Immunization data are available only up to 2022, unlike epidemiological and disease notification data, which are available through 2024.

Between 2010 and 2022, the vaccination coverage rates in Brazil followed a similar pattern across all five regions. After the vaccine was introduced into the PNI in 2010, coverage rose from 26% to over 95% the following year and remained near this level until 2015. A slight decline occurred until 2019, followed by a sharp drop during the COVID-19 pandemic, reaching the lowest rate since the vaccine's implementation in 2021; only 72% of children aged 3 months to 1 year were vaccinated (Table 1).

**Table 1:** Evolution of MenC vaccine coverage in Brazil from 2010 to 2022

Year	Vaccination coverage (%) by region of Brazil					
	North	Northeast	Southeast	South	Midwest	Total
2010	3.03	28.53	38.21	4.77	10.12	26.88
2011	78.71	93.41	115.51	121.63	151.10	105.66
2012	88.20	94.22	98.48	98.86	98.92	96.18
2013	89.76	96.30	102.13	103.84	107.12	99.70
2014	86.44	93.20	98.29	100.58	104.38	96.36
2015	87.19	97.40	100.81	101.48	97.35	98.19
2016	81.87	88.68	93.12	94.51	103.15	91.68
2017	78.58	85.66	89.65	92.13	86.87	87.44
2018	74.10	90.44	90.77	88.65	89.47	88.49
2019	84.23	86.32	86.67	93.40	88.89	87.41
2020	71.53	76.09	79.16	89.16	83.71	79.23
2021	66.06	69.41	71.77	81.54	76.15	72.17
2022	74.63	78.86	75.97	85.61	83.84	78.63

Source: prepared by the authors using data from Brasil. Ministério da Saúde. Departamento de Informática do SUS (DATASUS). Sistema de Informação do Programa Nacional de Imunizações (SI-PNI). Brasília (DF): Ministério da Saúde; [citado 2025 Ago 20]. Disponível em: http://tabnet.datasus.gov.br^(8)^

Table 2 presents the distribution of confirmed cases across different age groups, based on the years of symptom onset from 2010 to 2024. At the beginning of the analysis period, the total number of confirmed cases was high, peaking in 2012 (12,700 cases), followed by a progressive decline in the following years.

**Table 2:** Confirmed meningitis cases by age group (years) according to the year of the first symptom (2010–2024)

Year	Confirmed cases by age group (years)				
	0–1	1–4	5–9	10–14	Total
2010	3191	3621	2968	1888	11,668
2011	2891	3663	3213	1824	11,591
2012	2739	4224	3750	1987	12,700
2013	2629	3522	2992	1515	10,658
2014	2546	3135	2648	1317	9,646
2015	2758	2706	1779	1039	8,282
2016	2524	2923	1904	969	8,320
2017	2516	3195	2140	1126	8,977
2018	2865	3250	2114	1040	9,269
2019	2655	3181	1988	992	8,816
2020	1593	716	430	353	3,092
2021	1449	631	326	321	2,727
2022	1738	2348	1410	621	6,117
2023	2214	3153	2443	853	8,663
2024	666	522	426	245	1,859

Source: prepared by the authors using data from Brasil. Ministério da Saúde. Secretaria de Vigilância em Saúde. Sistema de Informação de Agravos de Notificação - SINAN. Brasília (DF): Ministério da Saúde; [citado 2025 Ago 20]. Disponível em: https://www.gov.br/saude/ptbr/acesso-a-informacao/acoes-e-programas/sinan^(7)^

From 2010 to 2015, annual case numbers ranged from 9,646 to 12,700. Starting in 2016, a steady decline was observed and was accentuated in 2020, possibly due to the COVID-19 pandemic. In 2021, the number of confirmed cases dropped to 2,727, the lowest in the series, followed by a slight rebound, reaching 6,117 in 2023. Partial data from 2024 indicated 1,859 cases, suggesting continued low levels.

Regarding the age group distribution, children aged 0-1 and 1-4 years accounted for the majority of cases throughout the analysis period. For instance, in 2012, 2,739 cases were recorded in the 0-1 year age group and 4,224 in the 1-4 year age group. This pattern has persisted in recent years, although at a lower absolute number. In 2023, there were 1,738 and 2,343 cases in these groups, respectively. The 10-14 age group consistently showed the lowest absolute case numbers, ranging from 1,888 cases in 2010 to 245 in 2024.

Based on table 3, there was a general trend of decreasing total deaths over the years, with higher values at the beginning of the historical series and a progressive decline from 2016 onward. The highest number of deaths occurred in 2010 (2,038), followed by slight fluctuations in subsequent years, reaching significantly lower levels from 2019 onward. In 2020, the total number of deaths dropped to 724, followed by a slight increase in the following years, although it was still at a lower level compared with the early period.

**Table 3:** Registered deaths by age group (years) according to the year of the first symptom (2010–2024)

Year	Confirmed deaths by age group (years)				
	0–1	1–4	5–9	10–14	Total
2010	301	253	161	114	2,038
2011	239	188	149	100	2,021
2012	201	137	117	119	1,852
2013	176	119	104	108	1,800
2014	179	105	94	75	1,616
2015	171	103	57	67	1,586
2016	176	98	55	56	1,513
2017	155	104	66	44	1,582
2018	152	97	59	44	1,508
2019	166	96	61	54	1,467
2020	88	46	19	35	724
2021	81	39	24	29	785
2022	98	100	54	53	1,349
2023	142	121	69	56	1,614
2024	43	38	24	19	536

Source: prepared by the authors using data from Brasil. Ministério da Saúde. Secretaria de Vigilância em Saúde. Sistema de Informação de Agravos de Notificação - SINAN. Brasília (DF): Ministério da Saúde; [citado 2025 Ago 20]. Disponível em: https://www.gov.br/saude/ptbr/acesso-a-informacao/acoes-e-programas/sinan^(7)^

When analyzing the age-specific distribution, the 0-1 and 1-4 age groups accounted for most deaths in all years of the analyzed period. For example, in 2010, there were 301 deaths in the 0-1 year age group and 253 deaths in the 1-4 year group. This pattern persisted in subsequent years, with a progressive decline in absolute numbers. In 2023, 142 and 121 deaths were recorded in these groups, respectively. The 10-14 age group consistently showed the lowest number of deaths, ranging from 114 in 2010 to 56 in 2023.

Furthermore, table 4 presents data on the number of confirmed meningitis cases by serogroup (C, A, W, and Y) between 2010 and 2024. Serogroup C had the highest number of cases throughout the study period, peaking in 2010 (1,213 cases) and progressively declining to 45 cases by 2024. Serogroups A, W, and Y showed significantly lower numbers. Serogroup A had few records until 2017, and data have not been recorded (NR) since 2019. Serogroup W peaked at 84 cases in 2011 and declined to six cases in 2024. Serogroup Y also showed a reduction from 38 cases in 2012 to eight in 2024.

**Table 4:** Confirmed meningitis cases by serogroup according to the year of the first symptom (2010–2024)

Year	Confirmed meningitis cases by serogroup			
	C	A	W	Y
2010	1,213	4	76	14
2011	1,145	2	84	31
2012	1,037	7	66	38
2013	726	5	77	26
2014	549	3	59	23
2015	360	3	53	13
2016	316	1	50	18
2017	367	2	53	16
2018	319	NR	54	18
2019	257	NR	41	22
2020	68	NR	10	10
2021	34	NR	4	4
2022	120	NR	12	15
2023	173	NR	19	21
2024	45	NR	6	8

Source: prepared by the authors using data from Brasil. Ministério da Saúde. Secretaria de Vigilância em Saúde. Sistema de Informação de Agravos de Notificação - SINAN. Brasília (DF): Ministério da Saúde; [citado 2025 Ago 20]. Disponível em: https://www.gov.br/saude/ptbr/acesso-a-informacao/acoes-e-programas/sinan^(7)^

NR: not reported.

**Figure 1:**
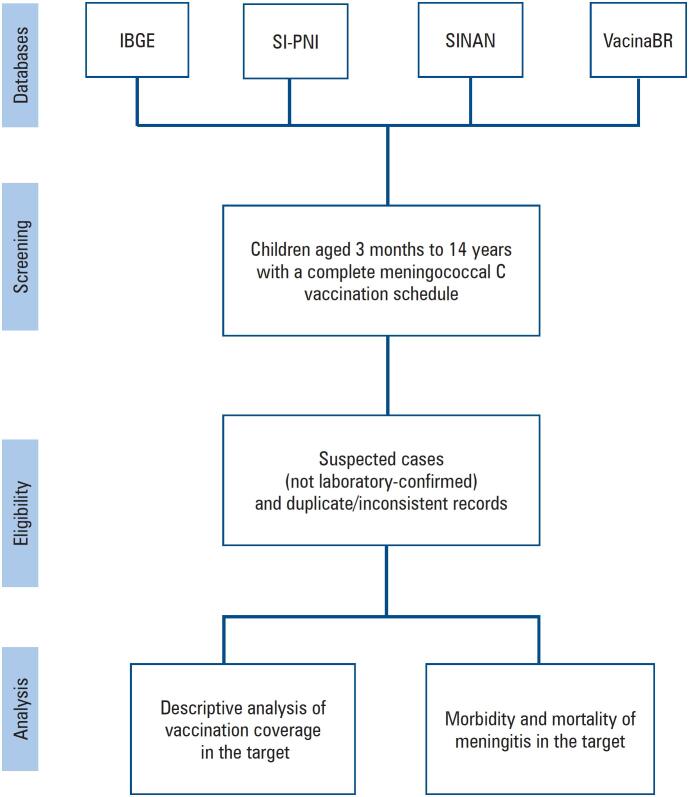
Flowchart of data selection and the analysis process

Figure 1 illustrates the relationship between vaccination coverage, confirmed cases, and meningitis-related deaths from meningitis C in children aged 0-14 years (2010-2022). Coverage increased rapidly until 2011 but declined after 2015, with a sharp drop after 2019. In parallel, cases and deaths decreased between 2010 and 2015, stabilizing at low levels from 2016 onward. During the pandemic (2020-2021), they reached their lowest values; however, in 2022, the incidence increased again, possibly because of decreased vaccination coverage (table 5). These data reinforce the association between high vaccination coverage and reduced mortality rates.

**Table 5:** Number of meningococcal conjugate C vaccine doses administered and nationwide coverage

Year	MenC vaccine doses administered					
	1	Coverage (%)	2	Coverage (%)	Booster	Coverage (%)
2010	1.338.598	48.52	639.104	22.33	707.831	24.56
2011	3.185.613	109.36	3.043.101	104.47	2.027.042	70.83
2012	2.784.283	95.82	2.769.917	95.33	2.453.963	84.24
2013	2.855.335	98.33	2.830.068	97.46	2.642.059	90.93
2014	2.840.351	95.34	2.798.991	93.95	2.569.112	88.47
2015	2.917.835	96.70	2.849.450	94.43	2.549.172	85.57
2016	2.760.554	96.60	2.728.674	95.48	2.793.126	92.56
2017	2.731.177	93.42	2.602.460	89.02	2.457.097	85.98
2018	2.647.498	89.90	2.525.656	85.77	2.289.582	78.32
2019	2.655.192	93.20	2.555.442	89.69	2.507.707	85.16
2020	2.429.712	89.00	2.316.150	84.84	2.237.928	78.55
2021	2.169.879	81.06	2.055.895	76.80	1.956.321	71.66
2022	2.243.448	87.57	2.130.029	83.18	2.048.817	76.53
2023	2.029.761	80.17	1.931.029	76.27	1.846.855	72.09
2024	NR	NR	NR	NR	NR	NR

Source: prepared by the authors using data from Brasil. Ministério da Saúde. Departamento de Informática do SUS (DATASUS). Sistema de Informação do Programa Nacional de Imunizações (SI-PNI). Brasília (DF): Ministério da Saúde; [citado 2025 Ago 20]. Disponível em: http://tabnet.datasus.gov.br^(8)^

NR: not reported.

## DISCUSSION

The present study revealed a substantial reduction in meningococcal C meningitis cases and related deaths in Brazil following the introduction of the meningococcal C vaccine into the PNI in 2010, with significant declines observed between 2010 and 2022. Analysis of vaccination coverage data showed that, despite successful nationwide implementation, the northern region encountered difficulties in reaching the 95% coverage target, underscoring persistent inequalities in access to immunization.^(1)^

Although the observed decrease in cases and mortality are primarily attributed to vaccination, these findings should be interpreted in light of evidence from similar international studies. Miller et al. and Koelman et al. reported significant reductions in the incidence of meningococcal C (up to 80% in the early years) in countries that adopted vaccination programs.^(13,14)^ In Brazil, herd immunity resulting from high vaccination coverage likely contributed to the reduction in cases among both vaccinated and unvaccinated individuals. This observation is consistent with the findings of Carvalheiro, who highlighted the indirect benefits of mass immunization strategies.^(15)^ The marked decline in mortality is also supported by international evidence, such as that of Wu et al., who reported a reduction in meningococcal C mortality in China following the implementation of a similar vaccination program.^(16)^

The resurgence of cases in 2022, particularly in specific regions, warrants further investigation. Regional disparities in the vaccine impact may reflect the underlying socioeconomic and structural challenges that compromise the effectiveness of public health initiatives. These findings are in line with the previous literature, which emphasizes the difficulties faced by areas with limited infrastructure, low levels of health education, and factors associated with increased vaccine hesitancy, as discussed by Arroyo et al. and Succi.^(17,18)^

Additionally, a descriptive ecological study analyzing meningitis cases and deaths in Brazil from 2010 to 2019 using the Notifiable Diseases Information System showed that spatial analyses revealed a higher prevalence and mortality in the south, southeast, and capital of Pernambuco, while higher case fatality rates were observed in the northern and northeastern regions, reflecting structural geographic and health inequities.^(19)^

The decline in vaccination coverage did not have an immediate impact on the incidence of severe cases, as the collective immunity acquired in previous years may offer residual population protection. However, interruptions or reductions in vaccination rates create favorable conditions for outbreak resurgence, especially among unvaccinated individuals or those with insufficient immune responses. Regarding *Neisseria meningitidis*, the etiological agent of meningococcal meningitis, decreased vaccination may lead to medium- and long-term increases in severe cases among children and adults, given the transmission of the bacterium via close contact and its facilitation in crowded environments.^(20)^ This phenomenon may be observed in the post-COVID-19 pandemic period, during which the health crisis impacted not only adherence to immunization campaigns but also meningococcal C transmission patterns.^(21)^

In this context, the present study encompassed the period coinciding with the COVID-19 pandemic, which may have influenced both the notification and incidence of new cases, as the dynamics of meningitis cases and deaths in recent years were strongly affected. The sharp reduction in records beginning in 2020 may be related to decreased healthcare-seeking behaviors and changes in reporting patterns. Fear of exposure to SARS-CoV-2 has also contributed to a decline in childhood vaccination, including meningococcal C, whereas social distancing may have temporarily reduced the circulation of *Neisseria meningitidis*. With the resumption of in-person activities, low vaccination coverage may have increased population vulnerability, contributing to the recent increase in cases and deaths, thereby highlighting the need for strategies to improve vaccination coverage and prevent outbreaks.^(22)^

This study confirms the effectiveness of the meningococcal C vaccine in controlling meningitis C in Brazil, underscoring the need for sustained strategies to reduce vaccine dropout, particularly in regions facing logistical and educational challenges. The use of updated nationally representative health data enhances the representativeness of the findings and enables a robust analysis of trends in cases and deaths considering variables such as the age group and vaccination coverage. Furthermore, identifying the impact of the COVID-19 pandemic on vaccination rates and disease incidence provides valuable insights for formulating public policy, particularly regarding equitable access, adherence to immunization schedules, and mitigation of future health crises.^(22)^

Nevertheless, it is important to acknowledge the inherent limitations of descriptive exploratory studies based on secondary data. Although national registry data are generally reliable, they may be subject to reporting errors or under-reporting, especially in remote areas of the country. The variability in the quality of information across health surveillance systems may affect the accuracy of vaccination coverage and disease incidence data. Additionally, this analysis raises hypotheses regarding unobserved variables such as shifts in population attitudes toward vaccination and the influence of public health campaigns, which may have impacted vaccination behavior in ways not captured by the available data.^(10)^

Despite the methodological limitations, this study makes several important contributions. The use of updated data allowed for the detailed monitoring of temporal trends in meningococcal C cases and deaths, enabling comprehensive analysis over time. Moreover, the integration of information from national epidemiological surveillance systems facilitated correlations among key variables, including the target vaccination age group, reinforcing the relevance of this study for public health policy development. The nationwide scope of the data, encompassing records across Brazil via the SUS, further strengthens the representativeness of the results and provides a broad perspective on the impact of the disease.^(6)^

## CONCLUSION

The introduction of the meningococcal C vaccine in 2010 markedly reduced the incidence and mortality of meningitis C in Brazil, demonstrating its effectiveness and early establishment of herd immunity. Nonetheless, vaccination coverage has declined since 2015, a trend worsened by the COVID-19 pandemic, with the resurgence of cases in 2022 signaling a weakening of collective protection. Persistent regional disparities, particularly in the north, reflect structural, social, and geographic inequities, creating pockets of susceptibility that undermine the equity and effectiveness of the national vaccination program.

This study highlights the need for targeted strategies to recover vaccination coverage, especially in vulnerable regions, and to integrate immunization with other child health services. Strengthening epidemiological surveillance through improved data quality and integration across national systems is essential. Future research should investigate the determinants of vaccine hesitancy, assess the long-term impact of declining coverage, and apply predictive modeling to estimate outbreak risks. By providing an updated and nationally representative overview from 2010 to 2024, these findings provide evidence to guide public policies, support equitable vaccination strategies, and enable international comparisons.

## Data Availability

The underlying content is contained within the manuscript.
